# Monthly Summary

**Published:** 1884-02

**Authors:** 


### MONTHLY SUMMARY.

---

DANGER—CARELESSNESS.—As the nature, extent, and influence of disease germs become more and better understood, does the responsibility of the dentist, as well as that of the general surgeon, become more apparent, in respect to the cleanliness and purity of his instruments and appliances of every kind. All are familiar with facts that ought, if at all considered, strongly impress the subject upon the mind of every one. For instance, an amount of small-pox virus, so small as to be scarccly visible when put upon a little scratch or abrasion in the skin, is sufficient to produce that dread disease throughout the body.

Every one knows, or ought to know, what is accomplished by an exceedingly small amount of vaccine virus.

The sting or bite of an isect is usually attended with very unpleasant results, such as pain, inflammation and sickness; the former two sometimes extending so as to involve other structures and organs, and sometimes these conditions are of long continuance. In many instances death has occurred from these accidents.

Now, in such cases, these results occur, usually from an amount of poison, invisible to the ordinary, unassisted eye. The poison from the knife of the dissector or surgeon, though not apparent upon the instrument, in many instances produces disastrous results, in the way of blood-poisoning ; many valuable lives have been lost by this means.

With knowledge of these and similar facts, how exceedingly cautious is the prudent surgeon, not only with reference to himself, but especially with his patients ; careful in reference to the purity of his instruments, his hands, and every appliance used ; and so important is this principle now regarded that the condition of the atmosphere receives special attention, hence the general use of disinfectants and anticeptics, that shall apply not only to the instruments and appliances, but pervade and purify the surrounding atmosphere.

If all this is important so far as the general surgeon is concerned, is it not equally important that the dentist should employ the same care and precaution? To the dentist, this should come with as much if not more impressiveness than to the general surgeon ; for the instruments and appliances of the dentist are passing in rapid succession from the diseased and wounded mouth of one patient to another, and it requires no special effort of the imagination to perceive that poison may thus be readily conveyed from one person to another, in the form of dead blood, decomposing pus, and other putrifying organic matter.

But is it possible that anybody is thus careless. Yes. In many offices there is a manifest want of cleanliness, which is shown by blood, pus, calculus, and other debris on the instruments—blood on the forceps, debris of decay, and other things on the excavators, comminuted dentine in the burs and drills, a spittoon with offensive fetid blood, and soiled napkins. Such condition of things should not be tolerated nor exist.

In the dental office too great care cannot be exercised in respect to cleanliness. All instruments used in the mouth should be thoroughly cleansed after use for each patient. This may be done by thorough washing in pure water and soap, then wipe with a cloth or chamois skin, slighly moistened with some disinfectant—a solution of salicylic acid. A very good solution for this purpose may be made as follows : One ounce water ; twenty grains borax, and twenty grains salicylic acid. This can be used without offensive taste or odor. The common sense of refinement, it would seem, should dictate to everyone the importance of absolute cleanliness. But this, in a general way, is not enough ; attention should constantly be given to the fact, that harmful agents are often present in an invisible form, and only by the utmost care can the best condition be maintained.

It may be said that these suggestions are superfluous. Well, in some instances they may be ; in many others, I know they are not, and some will never reform by anything that might be said ; but there are some—a few—who will regard good counsel, and the young more particularly will accept and adopt new and better ways.—*Dr. Taft in Dental Register.*

---

CHROMIC ACID AS A CAUSTIC.—Dr. Squibb recommends chromic acid as a most erosive caustic, as it is self-limiting in its action in a degree no other destructive caustic is. It is an active oxidixing agent, and destroys the tissues by oxidation, thus resembling some others caustics, nitric acid for instance. But every molecule of chromic acid which destroys a molecule of organic tissue is itself destroyed and rendered inert by being reduced to an inert oxide of chromium, and this principle and degree of self-limitation are not obtained by any other caustic. As the solution of chromic acid is the best form of applying it, and as this is wanted as strong as possible a very good plan is to add half its weight of water to the acid. This makes a clear solution, but on cooling some of the acid crystalizes out and serves to keep the solution fully saturated until it is used. The solution is best applied by means of a glass rod with smooth point. One part of muriatic acid diluted with two of water will remore the recent chromic acid stains but it is next to impossible to eradiate the old stains.—*Pacific Med. and Surg. Journal.*

